# Evaluation of the effect of tumour size on outcomes for patients undergoing adrenalectomy for phaeochromocytoma: international multicentre analysis

**DOI:** 10.1093/bjsopen/zraf133

**Published:** 2025-12-01

**Authors:** Steffane McLennan, Kevin Verhoeff, Alessandro Parente, Alynne Ribano, Yanbo Wang, Zhicheng Wang, Jiale Zhou, Xiaorong Wu, Yonghui Chen, Maciej Śledziński, Andrzej Hellmann, Marco Raffaelli, Francesco Pennestrì, Mark Sywak, Alexander J Papachristos, Fausto F Palazzo, Tae-Yon Sung, Byung-Chang Kim, Yu-mi Lee, Fiona Eatock, Hannah Anderson, Maurizio Iacobone, Daryl Gray, Richard C Chaulk, Vasilis Kosmoliaptsis, Nicola Colucci, Harry V M Spiers, Albertas Daukša, Ozer Makay, Yigit Turk, Hafize Basut Atalay, Els J M Nieveen van Dijkum, Anton F Engelsman, Isabelle Holscher, Gabriele Materazzi, Leonardo Rossi, Chiara Becucci, Susannah L Shore, Alison Waghorn, Claire Fung, Radu Mihai, Sabapathy P Balasubramanian, Arslan Pannu, Shuichi Tatarano, David Velázquez-Fernández, Julie A Miller, Hazel Serrao-Brown, Yufei Chen, Marco Stefano Demarchi, Reza Djafarrian, Helen Doran, Michael J Stechman, Helen Perry, Johnathan Hubbard, Cristina Lamas, Philippa Mercer, Janet MacPherson, Supanut Lumbiganon, María Calatayud, Felicia Alexandra Hanzu, Oscar Vidal, Marta Araujo-Castro, Cesar Minguez Ojeda, Theodosios Papavramidis, Pablo Rodríguez de Vera Gómez, Abdulaziz Aldrees, Tariq Altwjry, Nuria Valdés, Cristina Álvarez-Escola, Iñigo García Sanz, Concepción Blanco Carrera, Laura Manjón-Miguélez, Paz De Miguel Novoa, Mónica Recasens, Rogelio García Centeno, Cristina Robles Lázaro, Klaas Van Den Heede, Sam Van Slycke, Theodora Michalopoulou, Sebastian Aspinall, Ross Melvin, Joel Wen Liang Lau, Wei Keat Cheah, Man Hon Tang, Han Boon Oh, John Ayuk, Robert P Sutcliffe

**Affiliations:** Department of Surgery, Division of General Surgery, University of Alberta, Faculty of Medicine & Dentistry, Edmonton, Alberta, Canada; Department of Surgery, Division of General Surgery, University of Alberta, Faculty of Medicine & Dentistry, Edmonton, Alberta, Canada; Department of Surgery, Division of General Surgery, University of Alberta, Faculty of Medicine & Dentistry, Edmonton, Alberta, Canada; Department of Hepatopancreatobiliary and Liver Transplant Surgery, Queen Elizabeth Hospital, Birmingham, UK; Institute of Liver Studies, King’s College Hospital, London, UK; Faculty of Life Sciences and Medicine, School of Immunology and Microbial Sciences, The Roger Williams Institute of Liver Studies, King's College London, London, UK; Department of Surgery, Division of General Surgery, University of Alberta, Faculty of Medicine & Dentistry, Edmonton, Alberta, Canada; Department of Urology, The First Affiliated Hospital of Jilin University, Changchun, China; Department of Urology, The First Affiliated Hospital of Jilin University, Changchun, China; Department of Urology, Renji Hospital, School of Medicine, Shanghai Jiaotong University, Shanghai, China; Department of Urology, Renji Hospital, School of Medicine, Shanghai Jiaotong University, Shanghai, China; Department of Urology, Renji Hospital, School of Medicine, Shanghai Jiaotong University, Shanghai, China; Division of General, Endocrine and Transplant Surgery, Medical University of Gdańsk, Gdańsk, Poland; Division of General, Endocrine and Transplant Surgery, Medical University of Gdańsk, Gdańsk, Poland; UOC Chirurgia Endocrina e Metabolica, Fondazione Policlinico Universitario Agostino Gemelli IRCCS, Centro di Ricerca in Chirurgia Endocrina e dell'Obesità, Università Cattolica del Sacro Cuore, Rome, Italy; UOC Chirurgia Endocrina e Metabolica, Fondazione Policlinico Universitario Agostino Gemelli IRCCS, Centro di Ricerca in Chirurgia Endocrina e dell'Obesità, Università Cattolica del Sacro Cuore, Rome, Italy; Endocrine Surgical Unit, Royal North Shore Hospital, Northern Sydney Local Health District, Sydney, New South Wales, Australia; Endocrine Surgical Unit, Royal North Shore Hospital, Northern Sydney Local Health District, Sydney, New South Wales, Australia; Department of Endocrine Surgery, Hammersmith Hospital, London, UK; Department of Surgery, Asan Medical Center, University of Ulsan College of Medicine, Seoul, Korea; Department of Surgery, Asan Medical Center, University of Ulsan College of Medicine, Seoul, Korea; Department of Surgery, Asan Medical Center, University of Ulsan College of Medicine, Seoul, Korea; Department of Endocrine Surgery, Royal Victoria Hospital, Belfast, UK; Department of Endocrine Surgery, Royal Victoria Hospital, Belfast, UK; Endocrine Surgery Unit, Department of Surgery, Oncology and Gastroenterology, University of Padua, Padua, Italy; Department of Surgery, London Health Sciences Centre–Victoria Hospital, Western University, London, Ontario, Canada; Department of Surgery, London Health Sciences Centre–Victoria Hospital, Western University, London, Ontario, Canada; Department of Surgery, Addenbrooke's Hospital, Cambridge University Hospitals NHS Foundation Trust, Cambridge, UK; Department of Surgery, Addenbrooke's Hospital, Cambridge University Hospitals NHS Foundation Trust, Cambridge, UK; Department of Surgery, Addenbrooke's Hospital, Cambridge University Hospitals NHS Foundation Trust, Cambridge, UK; Department of Surgery, Lithuanian University of Health Sciences, Kaunas, Lithuania; Centre of Endocrine Surgery, Ozel Saglik Hospital, Izmir, Türkiye; School of Medicine, Aristoteleio University of Thessaloniki, Thessaloniki, Greece; Department of General Surgery, Division of Endocrine Surgery, Ege University Hospital, Izmir, Türkiye; Department of General Surgery, Division of Endocrine Surgery, Ege University Hospital, Izmir, Türkiye; Department of Surgery, Amsterdam UMC, University of Amsterdam, Cancer Center Amsterdam, Amsterdam, the Netherlands; Department of Surgery, Amsterdam UMC, University of Amsterdam, Cancer Center Amsterdam, Amsterdam, the Netherlands; Department of Surgery, Amsterdam UMC, University of Amsterdam, Cancer Center Amsterdam, Amsterdam, the Netherlands; Endocrine Surgery Unit, University Hospital of Pisa, Pisa, Italy; Endocrine Surgery Unit, University Hospital of Pisa, Pisa, Italy; Endocrine Surgery Unit, University Hospital of Pisa, Pisa, Italy; Department of Endocrine and Breast Surgery, Royal Liverpool and Broadgreen University Hospitals Trust, Liverpool, UK; Department of Endocrine and Breast Surgery, Royal Liverpool and Broadgreen University Hospitals Trust, Liverpool, UK; Department of Endocrine and Breast Surgery, Royal Liverpool and Broadgreen University Hospitals Trust, Liverpool, UK; Department of Endocrine Surgery, Churchill Cancer Centre, Oxford University Hospitals NHS Foundation Trust, Oxford, UK; Department of General Surgery, Sheffield Teaching Hospitals Foundation Trust, Sheffield, UK; Department of General Surgery, Sheffield Teaching Hospitals Foundation Trust, Sheffield, UK; Department of Urology, Graduate School of Medical and Dental Sciences, Kagoshima University, Kagoshima, Japan; Servicio de Cirugía Endocrina y Laparoscopia Avanzada, Departamento de Cirugía, Instituto Nacional de Ciencias Médicas y Nutrición Salvador Zubirán, Mexico City, Mexico; Endocrine Surgery Unit, The Royal Melbourne Hospital, Melbourne, Victoria, Australia; Endocrine Surgical Unit, Royal North Shore Hospital, Northern Sydney Local Health District, Sydney, New South Wales, Australia; Department of Surgery, Cedars-Sinai Medical Center, Los Angeles, California, USA; Department of Thoracic and Endocrine Surgery and Faculty of Medicine, University Hospitals of Geneva, Geneva, Switzerland; Department of Thoracic and Endocrine Surgery and Faculty of Medicine, University Hospitals of Geneva, Geneva, Switzerland; Department of Endocrine Surgery, Salford Royal Hospital, Salford, UK; Department of Endocrine Surgery, University Hospital Wales, Cardiff, UK; Department of Endocrine Surgery, University Hospital Wales, Cardiff, UK; Department of Endocrine Surgery, St Thomas’ Hospital, London, UK; Endocrinology and Nutrition Department, Complejo Hospitalario Universitario de Albacete, Albacete, Spain; Endocrine Surgical Unit, Department of General Surgery, Christchurch Hospital, Christchurch, New Zealand; Department of Anaesthesia, Christchurch Hospital, Christchurch, New Zealand; Department of Surgery, Division of Urology, Khon Kaen University, Khon Kaen, Thailand; Endocrinology and Nutrition Department, Hospital Universitario 12 de Octubre, Madrid, Spain; Endocrinology, Hospital Clinic University Barcelona, Barcelona, Spain; Department of Endocrine Surgery, Hospital Clinic Barcelona, Universitat de Barcelona, IDIBAPS, Barcelona, Spain; Department of Endocrinology and Nutrition, Hospital Universitario Ramón y Cajal, Madrid, Spain; Urology Department, Hospital Universitario Ramón y Cajal, Madrid, Spain; 1st Propaedeutic Department of Surgery, AHEPA University Hospital, Aristotle University of Thessaloniki, Thessaloniki, Greece; Endocrinology and Nutrition Department, Hospital Universitario Virgen de la Macarena, Sevilla, Spain; Department of Surgery, King Abdulaziz Medical City, Riyadh, Saudi Arabia; Department of Surgery, King Abdulaziz Medical City, Riyadh, Saudi Arabia; Department of Endocrinology and Nutrition, Hospital Universitario Cruces, Biobizkaia, Bizkaia CIBERDEM/CIBERER, Endo-ERN, Bilbao, Spain; Endocrinology and Nutrition Department, Hospital Universitario La Paz, Madrid, Spain; General and Digestive Surgery Department, Hospital Universitario de La Princesa, Madrid, Spain; Endocrinology and Nutrition Department, Hospital Universitario Príncipe de Asturias, Madrid, Spain; Endocrinology and Nutrition Department, Hospital Universitario Central de Asturias, Oviedo, Spain; Instituto de Investigación Sanitaria del Principado de Asturias (ISPA), Oviedo, Spain; Endocrinology & Nutrition Department, Hospital Clínico San Carlos, Madrid, Spain; Endocrinology and Nutrition Department, Institut Català de la Salut Girona, Girona, Spain; Endocrinology and Nutrition Department, Hospital Universitario Gregorio Marañón, Madrid, Spain; Endocrinology and Nutrition Department, Hospital Universitario de Salamanca, Salamanca, Spain; General and Endocrine Surgery, Onze-Lieve-Vrouw Hospital Aalst-Asse-Ninove, Aalst, Belgium; General and Endocrine Surgery, Onze-Lieve-Vrouw Hospital Aalst-Asse-Ninove, Aalst, Belgium; Department of Endocrinology and Nutrition, Joan XXIII University Hospital, Tarragona, Spain; Department of General Surgery, Aberdeen Royal Infirmary, Aberdeen, UK; Department of General Surgery, Aberdeen Royal Infirmary, Aberdeen, UK; Division of Breast and Endocrine Surgery, Department of General Surgery, Ng Teng Fong General Hospital, National University Health System, Singapore; Division of Breast and Endocrine Surgery, Department of General Surgery, Ng Teng Fong General Hospital, National University Health System, Singapore; Division of Breast and Endocrine Surgery, Department of General Surgery, Ng Teng Fong General Hospital, National University Health System, Singapore; Division of Breast and Endocrine Surgery, Department of General Surgery, Ng Teng Fong General Hospital, National University Health System, Singapore; Department of Endocrinology, Queen Elizabeth Hospital, Birmingham, UK; Department of Hepatopancreatobiliary and Liver Transplant Surgery, Queen Elizabeth Hospital, Birmingham, UK

## Abstract

**Background:**

Surgical resection is the standard treatment for phaeochromocytoma (PCC). Current guidelines recommend an open approach for large tumours due to the increased risk of complications. This study aimed to characterize surgical outcomes for large (≥ 6 cm) and small (< 6 cm) PCCs and to identify factors that may improve postoperative outcomes.

**Methods:**

This retrospective cohort study of patients undergoing adrenalectomy for PCC in 49 international centres between 2012 and 2022 compared patients with tumours < 6 cm in diameter and those with tumours ≥ 6 cm in diameter. Univariate, bivariate (dichotomous), and multivariate (multiple logistic and linear) analyses were used to evaluate outcomes and risk factors for complications. A secondary multivariable analysis evaluated factors, including operative approach, influencing outcomes for patients with tumours ≥ 6 cm. A 1:1 propensity score-matched (PSM) analysis was completed to control for age, sex, body mass index, and the Charlson Co-morbidity Index.

**Results:**

Of the 2301 patients included in the analysis, 598 (26.0%) had PCCs with a diameter ≥ 6 cm. Patients with tumours ≥ 6 cm had a higher incidence of severe (Clavien–Dindo grade ≥ IIIa) postoperative complications (11.2% *versus* 4.8%; *P* < 0.001). Multivariable analysis revealed that tumour size ≥ 6 cm was an independent predictor of any complications (odds ratio (OR) 1.93; *P* < 0.001). Subanalysis of patients with tumours ≥ 6 cm demonstrated that laparoscopic (OR 0.33; *P* < 0.001) and robotic (OR 0.40; *P* = 0.038) adrenalectomy were independently associated with less morbidity than an open approach. PSM analysis revealed a mean 276.0-ml higher blood loss (95% confidence interval (c.i.) 138.9 to 413.0 ml; *P* < 0.001) and 2.9-point higher Comprehensive Complication Index (95% c.i. 0.6 to 5.3; *P* = 0.015) for patients with tumours ≥ 6 cm compared with patients with PCCs < 6 cm in diameter. Optimal cut-off analysis revealed that a tumour diameter of ≥ 5.8 cm was associated with increased complications.

**Conclusion:**

Patients undergoing adrenalectomy for PCCs ≥ 6 cm have a higher risk of severe complications than patients with smaller tumours. Despite this increased risk in patients with large (≥ 6 cm) tumours, minimally invasive surgery was independently associated with a reduced likelihood of complications. This study supports a minimally invasive approach in patients with large PCCs.

## Teaser

Surgical resection is the standard treatment for phaeochromocytoma, but guidelines recommend an open approach for large (≥ 6 cm) tumours due to an increased risk of complications. This study shows that patients undergoing adrenalectomy for phaeochromocytomas ≥ 6 cm in diameter have a higher risk of severe complications than patients with smaller tumours. Despite this increased risk, minimally invasive surgery in patients with large tumours is independently associated with a reduced likelihood of complications.

## Introduction

Phaeochromocytoma (PCC) is a rare catecholamine-producing neuroendocrine tumour that arises from adrenomedullary chromaffin cells^1^. Operative management remains the first-line treatment for these tumours, with most resections now being completed with minimally invasive approaches (MIAs)^2–5^. Laparoscopic adrenalectomy (LA) remains the standard for the resection of adrenal tumours, including PCC, with robotic adrenalectomy (RA) emerging as an additional MIA with similar outcomes to LA^6–8^. Given the increased intraoperative risk for capsular injury, subsequent haemodynamic fluctuations, and potential intraoperative mortality secondary to catecholamine release, the role of MIA over an open approach (OA) has historically been controversial for larger PCCs^2,5,6,6,9–11^. Clinical practice guidelines (2014) from the Endocrine Society^12^ recommended an open surgical approach for PCCs with a diameter > 6 cm due to the increased perioperative risk. There is emerging evidence for the safety of MIA for large PCCs, with a recent review of 600 patients undergoing LA demonstrating similar perioperative complication rates for both large (≥ 6 cm) and small (< 6 cm) PCCs^2^. However, with limited formal evidence characterizing how the size of PCCs affects a patient’s perioperative course, there is ongoing controversy regarding the preferred approach for the resection of larger tumours.

The aims of the present international multicentre study were to characterize clinical outcomes for patients after adrenalectomy for large (≥ 6 cm) and small (< 6 cm) PCCs and to identify factors, such as operative approach, that may influence postoperative outcomes.

## Methods

### Study design

This is a retrospective nested cohort study from a large clinical cohort from 49 centres across 21 countries, as described previously^8,13^. Centres were identified based on literature published within the past 10 years in endocrine and adrenal surgery. Included patients underwent surgery between January 2012 and December 2022. Patient information was submitted by respective sites to a central prospective database; the information was anonymized retrospectively and did not change clinical care pathways. The lead centre (Queen Elizabeth Hospital, Birmingham, UK) received study approval as a clinical audit (Registration CARMS [Audit Registration and Management System of Queen Elizabeth Hospital Birmingham]-18769). Sites in other countries were responsible for registering this study with their local ethics review boards.

### Data collection

Clinical information, including age, sex, preoperative co-morbidities, and body mass index (BMI), was collected. The Charlson Co-morbidity Index was used to score the preoperative co-morbidity burden^14^. Patient physical status was assessed using American Society of Anesthesiologists (ASA) grades^15^. A preoperative diagnosis of PCC was made based on elevated plasma/urine catecholamine levels according to individual centre protocols combined with cross-sectional imaging (computed tomography or magnetic resonance imaging). Patients undergoing primary adrenalectomy for PCC, including those with locally advanced disease requiring nephrectomy or metastatic disease, were included. Patients undergoing revisional resection were excluded. Tumour-specific information collected included tumour size and laterality, along with any underlying genetic conditions.

### Study outcomes

The primary outcome of the present study was the complication rate after resection of small *versus* large PCC. In this study, small tumours were defined as those < 6 cm in diameter and large tumours were defined as those ≥ 6 cm in diameter based on the clinical practice guidelines from the Endocrine Society^12^. In patients with bilateral lesions, the diameter of the larger tumour was used.

Secondary outcomes included evaluation of operative approach and perioperative outcomes, including operative time, estimated blood loss, need for perioperative blood transfusion, and the rate of and reason for conversion to an open or hand-assisted procedure. Other intraoperative events were not collected. Postoperative secondary outcomes included care location, length of hospital stay (LOS), and readmission within 90 days. Finally, the cut-off value defining large tumours had been previously determined subjectively; therefore, an additional secondary outcome was the determination of the tumour size associated with an increased risk of postoperative complications.

### Definitions, operative approach, and perioperative management

Adrenalectomy was performed with either MIA, including LA or RA, or an OA. LA and RA were performed with either a transperitoneal approach (TPA) or a retroperitoneal approach (RPA) according to surgeon or centre preference. Operative time was defined as time from skin incision to closure. Conversion to open was defined as the need for laparotomy in a patient booked for LA or RA, or completing the procedure using a hand-assisted (HA) technique. Peri- and postoperative complications between the day of surgery and discharge were evaluated using the Clavien–Dindo (CD) classification system^16^. Complications were defined as any deviation from the normal postoperative course, and each was assigned a CD grade between I and V based on the invasiveness of treatment required, with severe complications defined as CD grade ≥ IIIa. Prolonged vasopressor support (> 24 h) for postoperative hypotension was considered a grade IVa complication based on previous studies^17,18^. The Comprehensive Complication Index^19^ (CCI) was used to describe the overall complication burden on a scale of 0–100 at discharge and 90 days and 12 months after surgery for all patients included in the cohort. The LOS was calculated as the time from the day of surgery to discharge. Readmission for any cause within 90 days of discharge were evaluated.

### Statistical analysis

Statistical analyses were completed using Stata 17 (StataCorp, College Station, TX, USA) and significance was defined as *P* < 0.05 for two-tailed hypothesis testing. Data were checked for normal distribution and are presented as the mean with standard deviation (s.d.); differences between groups were evaluated using analysis of variance (ANOVA). Categorical variables are presented as counts and percentages, with the significance of differences analysed using χ^2^ tests. Odds ratios (ORs) were used to present results from multivariable and propensity matched analyses and are presented with their 95% confidence interval (c.i.). No methods to impute or delete missing data were used in this study because all demographic and most outcome measures were complete for at least 95% of patients. The outcomes that were < 95% complete were operative time (78.7%), margin status (78.1%), estimated blood loss (66.7%), and postoperative hypotension (78.8%).

Unadjusted bivariate analysis of cohort demographics was assessed between patients with PCC diameter < 6 cm and ≥ 6 cm. Further analysis was completed with the cohort of patients with PCC ≥ 6 cm, ≥ 7 cm, ≥ 8 cm, and equal or greater to the calculated optimal cut-off value to evaluate factors (including operative approach) associated with complications in these higher-risk cohorts. Two multivariable models were used to control for demographic differences and evaluate factors independently associated with complications and the linear association with CCI. A univariate analysis was first conducted to identify clinically and statistically relevant outcomes (*P* < 0.10) to generate a preliminary main effects logistic regression model. The Wald test was used to determine significant variables (*P* < 0.05) contributing to the model. The variance inflation factor was used to evaluate collinearity with values > 10 prompting exclusion from the model. The Brier score and receiver operating characteristic curves were used to assess the goodness of fit of the models.

A propensity score-matched (PSM) assessment was also completed. Patients with PCC < 6 cm and ≥ 6 cm were matched 1 : 1 based on propensity scores using probit treatment modelling controlling for age, sex, BMI, and the Charlson Co-morbidity Index to evaluate the average treatment effect. Replacements were allowed with patients with PCC ≥ 6 cm being allowed to match more than once to patients with PCC < 6 cm to improve the propensity matching. The quality of the matching was analysed using a balance plot evaluating the difference in propensity scores between cohorts. Outcomes assessed included the CCI at discharge, operative time, estimated blood loss, total LOS, and the occurrence of any complication.

To establish an optimal size cut-off associated with complications, an area under the curve analysis evaluating tumour size and complications was performed using Bamber and Hanley confidence intervals. The product of the sensitivity and specificity was used to determine the optimal cut-off value for tumour size associated with complications (that is, the size cut-off with optimal sensitivity/specificity for determining complications) using the Liu method^20^.

## Results

### Baseline characteristics

In all, 2301 patients underwent adrenalectomy for PCC and were evaluated; of these, 598 (26.0%) had PCC ≥ 6 cm in diameter. Most patients (81.3%) underwent LA, with fewer undergoing RA (6.6%), a hand-assisted technique (0.4%), and OA (11.7%). Most MIAs used a TPA (66.6%; *versus* 31.7% using an RPA). Mean(s.d.) patient age was 51.8(15.3) years, and the median Charlson Co-morbidity Index was 2.1. In the cohort of patients with PCC < 6 cm, tumour diameter ranged from 0.6 to 5.9 cm, with a mean(s.d.) diameter of 3.5(1.2) cm. For patients with PCC ≥ 6 cm, tumour diameter ranged from 6.0 to 30.0 cm, and the mean(s.d.) diameter was 8.4(2.8) cm (*Table 1*). Information regarding the geographical distribution of the present study cohort is provided in *Table S1*.

**Table 1:** Baseline characteristics of the population in the international phaeochromocytoma database overall and in patients undergoing resection of large (≥ 6 cm) and small (< 6 cm) PCC separately

	Total cohort (*n* = 2301)	Unmatched cohort			PS-matched cohort		
		PCC < 6 cm (*n* = 1703)	PCC ≥ 6 cm (*n* = 598)	P*	PCC < 6 cm (*n* = 201)	PCC ≥ 6 cm (*n* = 418)	P*
Age (years), mean(s.d.)	51.8(15.3)	51.8(15.5)	51.7(14.9)	0.899	52.4(15.6)	51.9(15.1)	0.691
BMI (kg/m^2^), mean(s.d.)	25.7(4.9)	25.8(5.0)	25.5(4.6)	0.275	25.2(5.2)	25.3(4.3)	0.778
**Sex**							0.461
Male	1252 (54.4%)	930 (54.6%)	322 (53.8%)		115 (57.2%)	226 (54.1%)	
Female	1049 (45.6%)	773 (45.4%)	276 (46.2%)	0.747	86 (42.8%)	192 (45.9%)	
Charlson Co-morbidity Index, mean(s.d.)	2.1(1.9)	2.1(1.9)	2.1(1.9)	0.999	2.2(1.9)	2.4(1.9)	0.273
**ASA grade**				0.408			0.351
I	384 (16.7%)	292 (17.2%)	92 (15.4%)		28 (13.9%)	43 (10.3%)	
II	1202 (52.3%)	892 (52.4%)	310 (52.0%)		91 (45.3%)	214 (51.2%)	
III	667 (29.0%)	482 (28.3%)	185 (31.0%)		76 (37.8%)	153 (36.6%)	
IV	46 (2.0%)	37 (2.2%)	9 (1.5%)		6 (3.0%)	8 (1.9%)	
**Co-morbidities**				< 0.001			0.077
Type 2 diabetes	120 (9.2%)	85 (8.9%)	35 (10.3%)		13 (9.6%)	16 (5.9%)	
Hypertension	850 (65.4%)	657 (68.4%)	193 (56.9%)		92 (68.2%)	168 (62.2%)	
Both	329 (25.3%)	218 (22.7%)	111 (32.7%)		30 (22.2%)	86 (31.9%)	
Genetic condition (yes)	316 (14.0%)	245 (14.7%)	71 (12.1%)	0.124	32 (16.6%)	55 (13.5%)	0.313
**Surgical approach**				< 0.001			< 0.001
Open	266 (11.7%)	94 (5.5%)	172 (30.0%)		19 (9.5%)	132 (31.6%)	
Laparoscopic	1845 (81.3%)	1487 (87.6%)	358 (62.5%)		159 (79.1%)	249 (59.6%)	
Robotic	149 (6.6%)	113 (6.7%)	36 (6.3%)		22 (11.0%)	35 (8.4%)	
Hand-assisted	10 (0.4%)	3 (0.2%)	7 (1.2%)		1 (0.5%)	2 (0.5%)	
**Approach**				< 0.001			< 0.001
Transperitoneal	1505 (66.6%)	1079 (63.8%)	426 (75.0%)		131 (65.5%)	329 (79.3%)	
Retroperitoneal	716 (31.7%)	602 (35.6%)	114 (20.1%)		69 (34.5%)	73 (17.6%)	
Other	39 (1.7%)	11 (0.7%)	28 (4.9%)		0 (0.0%)	13 (3.1%)	
Tumour size (largest diameter; cm), mean(s.d.)	4.7(2.7)	3.5(1.2)	8.4(2.8)	<0.001	3.6(1.3)	8.4(2.7)	< 0.001
**Tumour location**				0.172			0.808
Right	1097 (49.0%)	847 (50.5%)	223 (44.2%)		89 (45.0%)	172 (42.2%)	
Left	1070 (47.8%)	780 (46.5%)	290 (51.5%)		101 (51.0%)	219 (53.7%)	
Bilateral	86 (3.8%)	59 (4.8%)	27 (3.5%)		8 (4.0%)	17 (4.2%)	
β-Adrenoceptor blockade	718 (33.9%)	516 (32.5%)	202 (38.3%)	0.014			
α-Adrenoceptor blockade	1993 (91.3%)	1501 (91.5%)	492 (90.4%)	0.607			
Admitted before surgery for i.v. fluids	735 (34.3%)	543 (33.8%)	192 (35.8%)	0.251			
Local invasion/planned nephrectomy	23 (1.2%)	5 (0.4%)	18 (3.7%)	< 0.001			
Extra-adrenal metastases	38 (1.7%)	16 (1.0%)	22 (4.0%)	< 0.001			

Values are n (%) unless otherwise stated. PCC, phaeochromocytoma; PS, propensity score; BMI, body mass index; s.d., standard deviation; ASA, American Society of Anesthesiologists; i.v., intravenous. *P values were evaluated with ANOVA and χ2 tests.

Comparisons of patient demographics between the small (< 6 cm) and large (≥ 6 cm) PCC groups (*Table 1*) revealed that the two groups were similar in terms of age (51.8 *versus* 51.7 years, respectively; *P* = 0.899), BMI (25.8 *versus* 25.8 kg/m^2^; *P* = 0.275), and the proportion of women (45.4% *versus* 46.2%, respectively; *P* = 0.747). The Charlson Co-morbidity Index and ASA grades were also similar between the two groups (*Table 1*). Patients with PCC ≥ 6 cm were more likely to be scheduled for an OA (30.0% *versus* 5.5%; *P* < 0.001) and with a TPA (75.0% *versus* 63.8%; *P* < 0.001). A higher proportion of patients with PCC ≥ 6 cm had local invasion and underwent a planned nephrectomy (3.7% *versus* 0.4%; *P* < 0.001) or had extra-adrenal metastases (4.0% *versus* 1.0%; *P* < 0.001) and required preoperative β-adrenoceptor blockade (38.3% *versus* 32.5%; *P* < 0.014; *Table 1*).

### Bivariate analysis of postoperative outcomes

Comparison of non-adjusted outcomes between patients with large (≥ 6 cm) and small (< 6 cm) PCC revealed no significant difference in the rates of rates of readmission within 90 days (1.6% *versus* 1.7%, respectively; *P* = 0.843; *Table 2*). The operative time was longer for patients with PCC ≥ 6 cm (161.5 *versus* 118.5 min; *P* < 0.001), who also experienced a higher incidence of perioperative blood transfusion (15.9% *versus* 2.7%; *P* < 0.001; *Table 2*). Patients with a large PCC planned for MIA were more likely to be converted to an OA than patients with a small PCC (5.1% *versus* 2.5%; *P* = 0.001). There was also a lower rate of negative resection margins in the large PCC *versus* small PCC group (91.7% *versus* 95.3%; *P* = 0.008). Postoperatively, patients with large PCC were more likely to require intensive care unit-level care (39.9% *versus* 34.3%; *P* = 0.012; *Table 2*). Patients with PCC ≥ 6 cm had a two-fold higher probability of having any complications during admission (33.5% *versus* 15.5%; *P* < 0.001), including severe complications (11.2% *versus* 4.8%; *P* < 0.001). LOS was longer for patients with a large PCC (6.7 *versus* 5.1 days, *P* < 0.001). There were no cases of intra or post-operative mortality in either group (*Table 2*).

**Table 2:** Clinical outcomes in all patients undergoing adrenalectomy and in those with large (≥ 6 cm) and small (< 6 cm) PCC separately

	Total cohort (*n* = 2301)	PCC < 6 cm (*n* = 1703)	PCC ≥ 6 cm (*n* = 598)	P*
Operative time (min), mean(s.d.)	129.3(75.3)	118.5(63.5)	161.4(96.0)	< 0.001
Estimated blood loss (ml), mean(s.d.)	168.8(700.9)	74.0(228.1)	441.7(1278.8)	< 0.001
Perioperative blood transfusion	134 (6.1%)	45 (2.7%)	89 (15.9%)	< 0.001
Converted to open/HA	77 (3.2%)	42 (2.5%)	35 (5.1%)	0.001
Negative resection margin	1734 (94.4%)	1317 (95.3%)	417 (91.7%)	0.008
**Reason for conversion**				0.029
Bleeding	102 (4.3%)	74 (4.4%)	28 (4.1%)	
Instability	9 (0.4%)	4 (0.2%)	5 (0.7%)	
Tumour size	11 (0.5%)	4 (0.2%)	7 (1.0%)	
Other†	24 (1.0%)	15 (0.9%)	9 (1.3%)	
**Postoperative location**				
Ward	1020 (45.3%)	797 (47.3%)	223 (39.3%)	
High-acuity monitoring unit	375 (16.6%)	272 (16.1%)	103 (18.2%)	0.013
ICU	805 (35.7%)	579 (34.3%)	226 (39.9%)	
Postoperative vasopressor at skin closure	468 (21.4%)	296 (18.0%)	172 (31.3%)	< 0.001
Postoperative hypotension beyond 24 h	127 (6.8%)	66 (4.8%)	61 (12.8%)	< 0.001
Any complications during hospital stay	455 (20.0%)	262 (15.5%)	193 (33.5%)	< 0.001
LOS (days), mean(s.d.)	5.5(4.3)	5.1(4.1)	6.7(4.8)	< 0.001
**CCI, mean(s.d.)**				
At discharge	5.7(13.0)	4.2(11.0)	10.1(16.9)	< 0.001
90 days	6.0(13.4)	4.6(11.5)	10.3(17.0)	
12 months	6.2(13.6)	4.7(11.6)	10.6(17.5)	
Severe complication (CD ≥ IIIa)	146(6.4%)	80(4.8%)	66(11.2%)	< 0.001
Severe complication excluding hypotension	37(1.9%)	18(1.2%)	19(3.7%)	< 0.001
Readmission within 90 days	37(1.6%)	28(1.7%)	9(1.6%)	0.843
Postoperative mortality (until hospital discharge)	0	0	0	

Values are n (%) unless otherwise stated. PCC, phaeochromocytoma; min, minutes; i.q.r., interquartile range; HA, hand-assisted; ICU, intensive care unit; LOS, length of hospital stay; CCI, Comprehensive Complication Index; CD, Clavien–Dindo. *P values were evaluated with ANOVA and χ2 tests.

### Demographic-adjusted postoperative outcomes

Factors associated with the occurrence of any complication were evaluated for the entire patient cohort in this study using multivariable logistic evaluation to control for differences in demographic characteristics (*Table 3*). Larger tumour size was an independent risk factor associated with increased complications (OR 1.93; 95% c.i. 1.43 to 2.60; *P* < 0.001), along with an increased Charlson Co-morbidity Index (OR 1.20; 95% c.i. 1.11 to 1.30; *P* < 0.001). Both LA (OR 0.30; 95% c.i. 0.21 to 0.43; *P* < 0.001) and RA (OR 0.38; 95% c.i. 0.21 to 0.68; *P* = 0.001) were associated with a reduced likelihood of complications. Patient age, sex, and BMI were not associated with complications. Similarly, when evaluating factors associated with increased CCI in a multivariable linear regression, larger tumour size and increased Charlson Co-morbidity Index were independently associated with increased CCI, whereas LA and RA were associated with a reduced CCI (*Table 3*).

**Table 3:** Multivariable analyses of risk factors for any complications at the time of discharge and for CCI in patients undergoing adrenalectomy

Risk factor	Odds ratio*	*P*
**Factors associated with any complication**		
Tumour size (large *versus* small†)	1.93 (1.43, 2.60)	< 0.001
Age	0.99 (0.98, 1.00)	0.083
Sex (female *versus* male)	0.98 (0.74, 1.28)	0.854
BMI ≥ 30 kg/m^2^	1.00 (0.97, 1.30)	0.846
Surgical approach (*versus* open)		
Laparoscopic	0.30 (0.21, 0.43)	< 0.001
Robotic	0.38 (0.21, 0.68)	0.001
Hand-assisted	0.39 (0.04, 4.31)	0.446
Charlson Co-morbidity Index	1.20 (1.11, 1.30)	< 0.001
Tumour laterality		
Left-sided tumour (*versus* right)	1.00 (0.76, 1.31)	0.758
Bilateral tumour	1.35 (0.69, 2.63)	0.384
Retroperitoneal approach (*versus* transperitoneal)	0.98 (0.74, 1.29)	0.866
Planned nephrectomy	1.68 (0.62, 4.57)	0.307
Brier score: 0.1345; ROC: 0.687		
**Factors associated with CCI**	Coefficient*	
Tumour size	3.32 (1.89, 4.76)	< 0.001
Age	−0.03 (−0.08, 0.02)	0.213
Sex (female *versus* male)	0.07 (−1.10, 1.23)	0.908
BMI ≥ 30 kg/m^2^	0.05 (−0.07, 0.18)	0.390
Surgical approach (*versus* open)		
Laparoscopic	−8.79 (−10.76, −6.82)	< 0.001
Robotic	−8.47 (−11.29, −5.65)	< 0.001
Hand-assisted	−2.96 (−14.76, 8.84)	0.623
Charlson Co-morbidity Index	0.65 (0.26, 1.03)	0.001
Tumour laterality		
Left-sided tumour (*versus* right)	0.09 (−1.09, 1.27)	0.879
Bilateral tumour (*versus* right)	1.57 (−1.58, 4.73)	0.329
Retroperitoneal approach (*versus* transperitoneal)	−0.32 (−1.55, 0.91)	0.609
Planned nephrectomy	6.10 (0.37, 11.82)	0.037
*R*^2^ = 0.0983		

^*^Values in parentheses are 95% confidence intervals. †Large and small tumours were defined as being ≥ 6 and < 6 cm, respectively. CCI, Comprehensive Complication Index; BMI, body mass index; ROC, receiver operating characteristic.

### PSM postoperative outcomes

Comparing patients with large and small PCCs in after 1 : 1 propensity score matching revealed no significant difference in LOS (*Table 4*). However, for patients with a large PCC, operative time was 30.3 min longer (95% c.i. 16.1 to 44.6 min; *P* < 0.001), with a higher average estimated blood loss of 276.0 ml (95% c.i. 138.9 to 413.0 ml; *P* < 0.001), than for patients with a small PCC. Total CCI was 2.9 points higher (95% c.i. 0.6 to 5.3 points; *P* = 0.015) with a 0.1% increased rate of any complications (95% c.i. 0.0 to 0.1; *P* = 0.095). Propensity score matching efficacy was satisfactory, as demonstrated by balance plot analysis (*Fig. S1*). When comparing these two matched cohorts, patients with larger tumours were more likely to undergo an open procedure (31.6% *versus* 9.5%) with a TPA (79.3% *versus* 65.5%) than those with smaller tumours, reflecting the full cohort demographics (*Table 1*).

**Table 4:** Average estimated treatment effect comparing patients with large (≥ 6 cm) and small (< 6 cm) phaeochromocytomas undergoing adrenalectomy following 1 : 1 propensity score matching

	Average estimated treatment effect*	*P*
Operative time	30.3 (16.1, 44.6)	<0.001
Estimated blood loss	276.0 (138.9, 413.0)	<0.001
Any complications during hospital stay	0.1 (0.0, 0.1)	0.095
Length of hospital stay	0.4 (−0.5, 1.3)	0.393
Total CCI	2.9 (0.6, 5.3)	0.015

^*^Values in parentheses are 95% confidence intervals. CCI, Comprehensive Complication Index.

### Subgroup analysis of patients with large PCCs

To better characterize specific risks associated with the resection of larger PCCs, patients with PCCs ≥ 6 cm (598), ≥ 7 cm (418), and ≥ 8 cm (305) were analysed independently. The results of the multivariable logistic regression to assess factors associated with increased complications for patients with PCCs ≥ 6 cm revealed similar results to the whole-group analysis (*Table 5*). LA (OR 0.33; 95% c.i. 0.20 to 0.54; *P* < 0.001) and RA (OR 0.40; 95% c.i. 0.17 to 0.95; *P* = 0.038) were associated with a reduced likelihood of complications compared with an OA for patients with PCC ≥ 6 cm. Increased Charlson Comorbidity Index was associated with an increased risk of complications (OR 1.19; 95% c.i. 1.03 to 1.37; *P* = 0.018). Multivariable linear regression to assess factors associated with CCI similarly demonstrated that an MIA was associated with a decreased CCI at the time of discharge (*Table 5*). Similar reductions in the likelihood of complications were seen in the subgroups of patients with PCC ≥ 7 and ≥ 8 cm (*Tables S2*, *S3*). Patients with large PCC were more likely to have higher levels of catecholamine secretion (*Table S4*).

**Table 5:** Multivariable logistic regression analysis of risk factors for any complications at the time of discharge and multivariable linear regression of risk factors for CCI in patients with PCC ≥ 6 cm undergoing adrenalectomy

Risk factor	OR*	*P*
**Multivariable logistic regression of factors associated with any complication**		
Age	1.01 (0.99, 1.03)	0.569
Sex (female *versus* male)	1.10 (0.70, 1.74)	0.682
BMI ≥30 kg/m^2^	1.00 (0.94, 1.05)	0.858
Surgical approach (*versus* open)		
Laparoscopic	0.33 (0.20, 0.54)	< 0.001
Robotic	0.40 (0.17, 0.95)	0.038
Hand-assisted†		
Charlson Co-morbidity Index	1.19 (1.03, 1.37)	0.018
Tumour laterality		
Left-sided tumour (*versus* right)	1.04 (0.65, 1.65)	0.870
Bilateral tumour	0.51 (0.13, 2.06)	0.344
Retroperitoneal approach (*versus* transperitoneal)	1.58 (1.00, 2.50)	0.049
Planned nephrectomy	1.69 (0.54, 5.22)	0.366
Brier score: 0.149; ROC: 0.614		
**Multivariable linear regression of factors associated with CCI**	Coefficient*	
Age	0.04 (−0.15, 0.22)	0.678
Sex (female *versus* male)	−0.72 (−4.97, 3.54)	0.740
BMI ≥ 30 kg/m^2^	0.28 (−0.28, 0.74)	0.376
Surgical approach (*versus* open)		
Laparoscopic	−8.45 (−12.91, −4.00)	< 0.001
Robotic	−9.28 (−18.28, −0.29)	0.043
Hand-assisted	−26.69 (−63.15, 9.77)	0.151
Charlson Co-morbidity Index	1.38 (0.01, 2.74)	0.048
Tumour laterality		
Left-sided tumour (*versus* right)	−1.53 (−5.87, 2.81)	0.488
Bilateral tumour (*versus* right)	−3.43 (−14.46, 7.59)	0.541
Retroperitoneal approach (*versus* transperitoneal)	1.17 (−3.12, 5.46)	0.592
Planned nephrectomy	3.48 (−6.72, 13.69)	0.502
*R* ^2^ = 0.1027		

^*^Values in parentheses are 95% confidence intervals. †Hand-assisted technique could not be evaluated. CCI, Comprehensive Complication Index; PCC, phaeochromocytoma; BMI, body mass index; ROC, receiver operating characteristic.

### Optimal cut-off value for PCC size to predict complications

An optimal cut-off value for PCC diameter to predict complications was determined to be 5.8 cm, with a sensitivity of 43.6% and a specificity of 79.0% (*Table S5*; *Fig. S2*). The positive likelihood ratio was 2.08 and the negative likelihood ratio was 0.71. The previously described cut-off of 6 cm had a sensitivity of 42.0% and a specificity of 79.8%. Lower cut-off values had a higher sensitivity, whereas higher cut-off values had a higher specificity (*Table S4*). Multivariable analysis revealed similar increases in the likelihood of complications for PCC ≥ 5.8 cm (OR 2.05; 95% c.i. 1.53 to 2.75; *P* < 0.001) compared with PCC ≥ 6 cm (*Table S6*).

## Discussion

This study represents the largest cohort available to evaluate the association of tumour size and clinical outcomes following adrenalectomy for PCC. The results show that operative time is longer, with a higher incidence of intraoperative blood transfusion and increased postoperative complications for patients with larger PCCs. The optimal PCC diameter to predict any complications was determined to be 5.8 cm. Despite the increased technical challenge with larger tumours, independent analysis of patients with large PCCs demonstrated that an MIA by both TPA and RPA is associated with a reduction in postoperative complications, regardless of size. These data highlight important safety outcomes and advocate for the consideration of minimally invasive techniques for patients with large PCC. In addition, given the higher complication rates, patients with large PCCs, and especially those with PCC ≥ 5.8 cm in diameter, may benefit from referral to high-volume centres with expertise in minimally invasive surgery.

Adrenalectomy for PCC is associated with a low mortality rate; however, peri- and postoperative complications are common^17^. Increased PCC size is associated with increased haemodynamic instability during surgery, with previous studies demonstrating increased risks with the resection of PCCs larger than 4–6 cm^5,12,21^. The analysis in the present study supports the findings of previous studies, demonstrating that patients with PCC ≥ 6 cm were over twice as likely to have any postoperative complication, including severe postoperative complications requiring reintervention. The analysis in the present study revealed that PCC ≥ 5.8 cm are associated with increased postoperative complications, which aligns with the previous arbitrarily defined cut-off of ≥ 6 cm^12^ .

Although, historically, OA has been recommended for large PCCs due to the increased risk of intraoperative capsular damage^12^ , the present study supports emerging evidence^2,5^ for the safety of an MIA for both large and small PCCs. Minimally invasive resection of PCC certainly requires considerable laparoscopic skills and expertise. The increased technical challenge of adrenalectomy for large PCC is reflected in the present data, with longer operative times and increased estimated blood loss requiring perioperative blood transfusions. Tumour seeding and recurrence in the adrenal bed have also been identified as potential challenges with LA^12,22^. In the present study, the whole-cohort analysis did demonstrate an increased incidence of positive margins following adrenalectomy for PCCs ≥ 6 cm, but the absolute difference in the rate of margin positivity was only 3.6%. With advances in laparoscopic surgery and the routine inclusion of these techniques within surgical training programs, it is not surprising that clinical outcomes for MIAs have improved over time. In addition to LA, RA for PCC is emerging as a safe and effective MIA and the present analysis supports the safety of this even for larger PCCs^8^. It is noteworthy that all centres contributing to this study were high-volume centres with extensive expertise in treating PCC; therefore, good outcomes could be achieved, even in the case of large tumours. Given the significantly increased risk of complications and the rarity of these tumours, referral to centres with high volumes and expertise in the resection of PCCs should be considered for all PCCs, especially those ≥ 5.8 cm in diameter.

Nearly two-thirds of adrenalectomies included in this study were completed via TPA. Due to the anatomical challenge of left-sided LA, a laparoscopic RPA was developed and recommended for benign adrenal tumours under 6 cm^23,24^. A meta-analysis^25^ from 2021 evaluating 775 patients undergoing adrenalectomy, including 196 patients with PCC, demonstrated that a RPA may be associated with a decrease in estimated blood loss and shorter LOS for PCC smaller than 6 cm; however, the safety of this approach has not been previously evaluated for patients with large PCC. It has been theorized that the smaller working space available with a RPA may limit its utility^25^; however, the data in the present study demonstrate that an RPA was not associated with increased complications compared with a TPA, even for large PCC.

Finally, the increased incidence of complications for patients with a PCC ≥ 5.8 cm in diameter presents an opportunity to risk stratify these patients before surgery. In the present study population, over 90% of patients were prescribed preoperative α-adrenoceptor blockade regardless of PCC size; however, patients with larger PCC were more likely to require adjunct antihypertensive therapies, such as β-adrenoceptor blockade. This raises the possibility of increased difficulty attaining preoperative normotension for patients with large PCC. Increasing PCC size has previously been shown to positively correlate with plasma (nor)metanephrines^26^, which is supported by the present data and likely contributes to this challenge. Interestingly, recent evidence^27^ suggests that hypertensive patients with PCC have a higher risk of postoperative complications than normotensive patients, but this seems to be related to a higher burden of co-morbidities and older age rather than hypertension. In addition, a meta-analysis showed that preoperative blockade of α-adrenoceptors may not improve intraoperative haemodynamic fluctuations or improve perioperative outcomes for PCC patients^28^; this is certainly an area that requires ongoing study to challenge current dogma. Considering the potential for increased complexity in the preoperative medical management of these patients, this would again support their management within centres with expertise in adrenalectomy for PCC.

The limitations of the present study are largely related to the retrospective nature of the data, and the results may thus be biased by unmeasured confounders. Because the data were collected from 49 centres with different healthcare systems, population demographics, and surgical techniques and training, differences in practice patterns may confound the data. There are many patient- and disease-related factors that may influence the decision to proceed with or convert to an OA and may not have been captured within the study data. PCC is a rare entity with unique technical considerations for resection given the potential for intraoperative haemodynamic effects; as such, the present data may not be applicable to other groups of patients who undergo adrenalectomy for other indications. In addition, intraoperative events were not available in the present cohort, limiting further analyses. There may also be an element of selection bias in the data in that the surgeons involved in the study are likely to have more experience with the resection of PCCs, and thus the data may not extrapolate well to centres that do not often perform adrenalectomy for PCC. Despite these limitations, the heterogeneity within the data provides a real-world evaluation of this cohort of patients, who have not been previously well characterized. The effects of confounding and demographic differences between groups were minimized by the use of multivariable modelling and PSM analysis.

The data demonstrate that the current cut-off of 6 cm for an MIA is outdated and does not reflect the standard of care at large referral centres internationally. Based on this study, representing the largest evaluation of PCC available internationally, MIA appears to benefit patients even with very large tumours. Considering this, it is suggested that a cut-off for tumour sizes recommended for an OA may not be clinically applicable with ongoing improvements in MIAs. However, future studies may evaluate whether specific approaches (robotic or retroperitoneal) appear superior for the largest PCCs or whether any specific learning curve exists for such tumours.

## Supplementary Material

zraf133_Supplementary_Data

## Data Availability

The authors confirm that the data supporting the findings of this study are available within the article and the *Supplementary material*. Raw data that support the findings of this study are available from the corresponding author upon reasonable request.
