# Supplementary material for: Evaluation of the effect of tumour size on outcomes for patients undergoing adrenalectomy for phaeochromocytoma: international multicentre analysis
Source: BJS Open. 2025 Dec 1;9(6):zraf133. doi: 10.1093/bjsopen/zraf133 (PMC12667028; doi:10.1093/bjsopen/zraf133)
Supplement: zraf133_Supplementary_Data [file zraf133_supplementary_data.docx]

**Evaluation of tumor size on outcomes for patients undergoing adrenalectomy for pheochromocytoma: International Multicenter Analysis**

Steffane McLennan MD, MSc^1^, Kevin Verhoeff MD, PhD^1^, Alessandro Parente MD, PhD^1,2,3^, Alynne Ribano^1^, Yanbo Wang MD, PhD^4^, Zhicheng Wang MD, PhD^4^, Jiale Zhou MD^5^, Xiaorong Wu MD^5^, Yonghui Chen MD, PhD^5^, Maciej Śledziński MD, PhD^6^, Andrzej Hellmann MD, PhD^6^, Marco Raffaelli MD, PhD^7^, Francesco Pennestrì MD^7^, Mark Sywak PhD^8^, Alexander J Papachristos MD^8^, Fausto F. Palazzo MD^9^, Tae-Yon Sung MD, PhD^10^, Byung-Chang Kim MD, PhD^10^, Yu-mi Lee MD, PhD^10^, Fiona Eatock MD^11^, Hannah Anderson MD^11^, Maurizio Iacobone MD^12^, Daryl Gray MD, PhD^13^, Richard C. Chaulk MD^13^, Vasilis Kosmoliaptsis MD, PhD^14^, Nicola Colucci MD^14^, Harry VM Spiers MD^14^, Albertas Daukša MD, PhD^15^, Ozer Makay MD^16^, Yigit Turk MD^17^, Hafize Basut Atalay MD^17^, Els J. M. Nieveen van Dijkum MD, PhD^18^, Anton F. Engelsman MD, PhD^18^, Isabelle Holscher MD^18^, Gabriele Materazzi MD, PhD^19^, Leonardo Rossi MD, PhD^19^, Chiara Becucci MD^19^, Susannah L. Shore MD^20^, Alison Waghorn MD^20^, Claire Fung MD^20^, Radu Mihai MD^21^, Sabapathy P. Balasubramanian PhD^22^, Arslan Pannu MD^22^, Shuichi Tatarano MD, PhD^23^, David Velázquez-Fernández MD, PhD^24^, Julie A Miller MD, PhD^25^, Hazel Serrao-Brown MD^8^, Yufei Chen MD, PhD^26^, Marco Stefano Demarchi MD, PhD^27^, Reza Djafarrian MD^27^, Helen Doran MD^28^, Michael J Stechman MD^29^, Helen Perry MD^29^, Johnathan Hubbard MD^30^, Cristina Lamas PhD^31^, Philippa Mercer MD^32^, Janet MacPherson MD^33^, Supanut Lumbiganon MD^34^, María Calatayud MD^35^, Felicia Alexandra Hanzu MD^36^, Oscar Vidal MD^37^, Marta Araujo-Castro MD, PhD^38^, Cesar Minguez Ojeda MD^39^, Theodosios Papavramidis MD, PhD^40^, Pablo Rodríguez de Vera Gómez MD^41^, Abdulaziz Aldrees MD^42^, Tariq Altwjry MD^42^, Nuria Valdés MD, PhD^43^, Cristina Álvarez-Escola MD, PhD^44^, Iñigo García Sanz MD^45^, Concepción Blanco Carrera MD^46^, Laura Manjón-Miguélez MD^47^, Paz De Miguel Novoa MD^48^, Mónica Recasens MD^49^, Rogelio García Centeno MD^50^, Cristina Robles Lázaro MD^51^, Klaas Van Den Heede MD, PhD^52^, Sam Van Slycke MD^52^, Theodora Michalopoulou MD^53^, Sebastian Aspinall MD^54^, Ross Melvin MD^54^, Joel Wen Liang Lau MBBS, PhD^55^, Wei Keat Cheah MBBS, PhD^55^, Man Hon Tang MBBS, PhD^55^, Han Boon Oh MBBS, PhD^55^, John Ayuk MD^56^, Robert P. Sutcliffe MA, MD^2^, On behalf of the International Pheo Study Group

**Affiliations:**

1 – Department of Surgery, Division of General Surgery, University of Alberta, Faculty of Medicine & Dentistry, Edmonton, Alberta, Canada.

2 - Department of Hepatopancreatobiliary and Liver Transplant Surgery, Queen Elizabeth Hospital, Birmingham, United Kingdom.

3 – Institute of Liver Studies, King’s College Hospital, Denmark Hill, SE59RS, London, United Kingdom. Roger Williams Institute of Liver Studies, Faculty of Life Sciences and Medicine, King's College London, United Kingdom.

4 - Department of Urology, The First Affiliated Hospital of Jilin University, Changchun, Jilin, China.

5 - Department of Urology, Renji Hospital, School of Medicine, Shanghai Jiaotong University, Shanghai, China.

6 - Division of General, Endocrine and Transplant Surgery, Medical University of Gdańsk, Poland.

7 - UOC Chirurgia Endocrina e Metabolica, Fondazione Policlinico Universitario Agostino Gemelli IRCCS, Centro di Ricerca in Chirurgia Endocrina e dell'Obesità, Università Cattolica del Sacro Cuore, Rome, Italy.

8 - Endocrine Surgical Unit, Royal North Shore Hospital, Northern Sydney Local Health District, Sydney, NSW, Australia.

9 - Department of Endocrine Surgery, Hammersmith Hospital, London, United Kingdom.

10 - Department of Surgery, Asan Medical Center, University of Ulsan College of Medicine, Seoul, 05505, Republic of Korea.

11 - Department of Endocrine Surgery, Royal Victoria Hospital, Belfast, United Kingdom.

12 - Endocrine Surgery Unit, Department of Surgery, Oncology and Gastroenterology, University of Padua, 35143 Padua, Italy.

13 - Department of Surgery, London Health Sciences Centre-Victoria Hospital, Western University, London, Ontario, Canada.

14 - Department of Surgery, Addenbrooke's Hospital, Cambridge University Hospitals NHS Foundation Trust, Cambridge, United Kingdom

15 - Department of Surgery, Lithuanian University of Health Sciences, Kaunas, Lithuania.

16 - Ozel Saglik Hospital, Centre of Endocrine Surgery, Izmir, Turkiye & School of Medicine, Aristoteleio University of Thessaloniki, Thessaloniki, Greece

17 - Ege University Hospital, Department of General Surgery, Division of Endocrine Surgery, Izmir, Türkiye

18 - Department of Surgery, Amsterdam UMC, University of Amsterdam, Cancer Center Amsterdam, Amsterdam, The Netherlands.

19 - Endocrine Surgery Unit, University Hospital of Pisa, Pisa, Italy.

20 - Department of Endocrine and Breast Surgery, Royal Liverpool and Broadgreen University Hospitals Trust, Liverpool, United Kingdom.

21 - Department of Endocrine Surgery, Churchill Cancer Centre, Oxford University Hospitals NHS Foundation Trust, United Kingdom.

22 - Department of General Surgery, Sheffield Teaching Hospitals Foundation Trust, Sheffield, United Kingdom.

23 - Department of Urology, Graduate School of Medical and Dental Sciences, Kagoshima University, Kagoshima, Japan.

24 - Servicio de Cirugía Endocrina y Laparoscopia Avanzada, Departamento de Cirugía, Instituto Nacional de Ciencias Médicas y Nutrición Salvador Zubirán, Mexico City, Mexico.

25 - Endocrine Surgery Unit, The Royal Melbourne Hospital, Melbourne, VIC, Australia.

26 - Cedars-Sinai Medical Center, Department of Surgery, Los Angeles, CA, United States.

27 - Department of Thoracic and Endocrine Surgery and Faculty of Medicine, University Hospitals of Geneva, 4 Rue Gabrielle Perret-Gentil, 1211 Geneva, Switzerland.

28 - Department of Endocrine Surgery, Salford Royal Hospital, Salford, United Kingdom.

29 - Department of Endocrine Surgery, University Hospital Wales, Cardiff, United Kingdom.

30 - Department of Endocrine Surgery, St Thomas’ Hospital, London, United Kingdom.

31 - Endocrinology & Nutrition Department. Complejo Hospitalario Universitario de Albacete, Albacete. Spain.

32 - Endocrine Surgical Unit, Department of General Surgery, Christchurch Hospital, New Zealand.

33 - Department of Anaesthesia, Christchurch Hospital. New Zealand.

34 - Department of Surgery, Division of Urology, Khon Kaen University, Khon Kaen, Thailand.

35 - Endocrinology & Nutrition Department. Hospital Universitario 12 de Octubre. Madrid. Spain.

36 - Endocrinology Hospital Clinic University Barcelona, Barcelona, Spain.

37 - Department of Endocrine Surgery, Hospital Clinic Barcelona. Universitat de Barcelona. IDIBAPS, Barcelona, Spain.

38 - Department of Endocrinology & Nutrition, Hospital Universitario Ramón y Cajal Madrid, Spain.

39 - Urology Department, Hospital Universitario Ramón y Cajal, Madrid, Spain

40 - 1st Propedeutic Department of Surgery, AHEPA University Hospital, Aristotle University of Thessaloniki, Thessaloniki, Greece.

41 - Endocrinology & Nutrition Department. Hospital Universitario Virgen de la Macarena. Sevilla. Spain.

42 - King Abdulaziz Medical City, Riyadh, Saudi Arabia.

43 - Department of Endocrinology and Nutrition, Hospital Universitario Cruces, Biobizkaia, Bizkaia CIBERDEM/CIBERER, Endo-ERN, Spain.

44 - Endocrinology & Nutrition Department. Hospital Universitario La Paz Madrid, Spain.

45 - General & Digestive Surgery Department. Hospital Universitario de La Princesa. Madrid, Spain.

46 - Endocrinology & Nutrition Department. Hospital Universitario Príncipe de Asturias. Madrid, Spain.

47 - Endocrinology & Nutrition Department. Hospital Universitario Central de Asturias. Oviedo, Spain & Instituto de Investigación Sanitaria del Principado de Asturias (ISPA). Oviedo, Spain

48 - Hospital Clínico San Carlos, Madrid, Spain.

49 - Endocrinology & Nutrition Department. Institut Català de la Salut Girona. Girona. Spain.

50 - Endocrinology & Nutrition Department. Hospital Universitario Gregorio Marañón. Madrid. Spain

51 - Endocrinology & Nutrition Department. Hospital Universitario de Salamanca. Salamanca, Spain.

52 - General and Endocrine Surgery, Onze-Lieve-Vrouw (OLV) Hospital Aalst-Asse-Ninove, Aalst, Belgium

53 - Department of Endocrinology and Nutrition, Joan XXIII University Hospital, Tarragona, Spain.

54 - Department of General Surgery, Aberdeen Royal Infirmary, Aberdeen, United Kingdom.

55 - Division of Breast & Endocrine Surgery, Department of General Surgery, Ng Teng Fong General Hospital (NTFGH), National University Health System (NUHS), Singapore.

56 - Department of Endocrinology, Queen Elizabeth Hospital, Birmingham, United Kingdom.

**Corresponding author**:

Alessandro Parente, MD, PhD, FEBS

Consultant Liver Transplant, Hepatobiliary and Pancreatic Surgeon

Honorary Senior Lecturer

Roger Williams Institute of Liver Studies, Faculty of Life Sciences and Medicine,

King's College London and King's College Hospital, London, UK.

Email: [alessandro.parente@kcl.ac.uk](mailto:alessandro.parente@kcl.ac.uk)

ORCID ID: 000-0001-5506-224X

**Supplementary Materials – Index**

| **Supplementary Figures and Tables** |  |
| --- | --- |
| Supplementary Figure 1  Balance plot of propensity scores demonstrating efficacy of 1:1 propensity matched cohort for patients undergoing adrenalectomy for PCC | *page 6* |
| Supplementary Table 1  Multivariable logistic regression for any complications at time of discharge for patients undergoing adrenalectomy for pheochromocytoma ≥ 7cm and ≥ 8cm | *page 7* |
| Supplementary Table 2  Multivariable logistic regression for any complications at time of discharge for patients undergoing adrenalectomy for pheochromocytoma ≥ 7cm and ≥ 8cm | *Page 9* |
| Supplementary Table 3  Multivariable linear regression for Comprehensive Complication Index (CCI) at time of discharge for patients undergoing adrenalectomy for pheochromocytoma ≥ 7cm and ≥ 8cm | *page 10* |
| Supplementary Table 4  Catecholamine quartiles for patients undergoing adrenalectomy for PCC | *page 11* |
| Supplementary Table 5  Sensitivity and specificity for predicting complications by PCC size for optimal cutoff analysis | *page 12* |
| Supplementary Figure 2  Receiver operating characteristic (ROC) curve demonstrating linearity of the sensitivity and specificity for any complications for patients undergoing adrenalectomy for PCC. | *page 13* |
| Supplementary Table 6  Multivariable logistic regression for any complications at time of discharge and multivariable linear regression for Comprehensive Complication Index (CCI) for patients undergoing adrenalectomy | *page 14* |
|  |  |

**Supplementary Figure 1.** Balance plot of propensity scores demonstrating efficacy of 1:1 propensity matched cohort for patients undergoing adrenalectomy for PCC

**Supplementary Table 1.** Geographic distribution of reported patients undergoing adrenalectomy for pheochromocytoma.

| Country | Centre(s) | Number of patients reported (n=2,303)* |
| --- | --- | --- |
| Australia | Royal Melbourne Hospital  RNSH University of Sydney | 141 |
| Belgium | Aalst OLV Ziekenhuis | 9 |
| Canada | London Health Sciences Centre | 71 |
| China | Jilin University Hospital  Shanghai Jiaotong University | 478 |
| Greece | Thessaloniki – Aristotle University | 14 |
| Italy | Fondazione Policlinico Universitario A Gemelli IRCCS  University Hospital of Pisa | 250 |
| Japan | Kagoshima University | 46 |
| Kingdom of Saudi Arabia | King Abdulaziz Medical City, Riyadh | 13 |
| Lithuania | Kaunas Lithuanian University of Health Sciences | 71 |
| Mexico | Instituto Nacional de Ciencias Médicas y Nutrición Salvador Zubirán, Mexico City | 42 |
| Netherlands | University of Amsterdam UMC | 65 |
| New Zealand | Christchurch Hospital, Canterbury | 18 |
| Poland | Medical University of Gdańsk | 104 |
| Singapore | Ng Teng Fong General Hospital | 6 |
| South Korea | University of Ulsan College of Medicine, Seoul | 87 |
| Spain | Hospital Universitario de Albacete, Albacete  Hospital Universitario Doce de Octubre, Madrid  Hospital Universitario Ramón y Cajal, Madrid  Hospital Clinic, Barcelona  Hospital Universitario Virgen de la Macarena, Sevilla  Hospital Universitario de Cabueñes, Asturias  Hospital Universitario La Paz, Madrid  Hospital Universitario de la Princesa, Madrid  Hospital Universitario Príncipe de Asturias, Madrid  Hospital Universitario Príncipe de Asturias, Oviedo  Hospital Clínico San Carlos, Madrid  Institut Català de la Salut Girona, Girona  Hospital Universitario Gregorio Marañón, Madrid  Hospital Universitario de Salamanca, Salamanca  Joan XXIII University Hospital, Tarragona | 197 |
| Switzerland | University Hospital of Geneva, Geneva | 33 |
| Thailand | Khon Kaen University, Khon Kaen | 17 |
| Turkey | Ege University Hospital, Izmir | 66 |
| United Kingdom | Hammersmith Hospital, London  Queen Elizabeth University Hospital, Birmingham  Royal Victoria Hospital, Belfast  Cambridge University Hospital, Cambridge  Royal Broadgreen Hospital, Liverpool  Oxford Hospital, Oxford  Sheffield Teaching Hospital, Sheffield  Salford Royal Hospital, Salford  University Hospital Wales, Cardiff  St. Thomas’ Hospital, London  Aberdeen Royal Infirmary, Aberdeen | 519 |
| US | Cedars Sinai Medical Centre, Los Angeles | 36 |

*the total number of reported patients was 2,303, however, 2 patients were removed from our analysis due to substantial data loss

**Supplementary Table 2.** Multivariable logistic regression for any complications at time of discharge for patients undergoing adrenalectomy for pheochromocytoma ≥ 7cm and ≥ 8cm

| **Multivariable Logistic Regression Evaluating Factors Associated with Any Complication for pheochromocytoma ≥ 7cm** | | | |
| --- | --- | --- | --- |
|  | |  | |
| Risk Factor | Odds Ratio | 95% Confidence Interval | p-value |
| Age | 1.02 | 0.99 – 1.05 | 0.258 |
| BMI | 0.95 | 0.88 – 1.03 | 0.203 |
| Sex | 0.73 | 0.38 – 1.41 | 0.341 |
| Charlson Comorbidity Index | 1.13 | 0.92 – 1.40 | 0.238 |
| Tumor laterality |  |  |  |
| Left sided tumor (compared to right) | 0.74 | 0.39 – 1.42 | 0.366 |
| Bilateral tumor | 0.57 | 0.11 – 2.92 | 0.499 |
| Retroperitoneal | 2.48 | 1.25 – 4.90 | 0.009 |
| Surgical Approach (compared to open) |  |  |  |
| Laparoscopic | 0.39 | 0.20 – 0.75 | 0.009 |
| Robotic | 0.39 | 0.10 – 1.55 | 0.183 |
| Hand Assisted | - | - | - |
| Planned nephrectomy | 0.90 | 0.25 – 3.25 | 0.869 |
| Brier Score: 0.1802  ROC: 0.5625 |  |  |  |
| **Multivariable Logistic Regression Evaluating Factors Associated with Any Complication for pheochromocytoma ≥ 8cm** | | | |
|  | |  | |
| Risk Factor | Odds Ratio | 95% Confidence Interval | p-value |
| Age | 1.04 | 0.99 – 1.09 | 0.136 |
| BMI | 0.93 | 0.82 – 1.06 | 0.288 |
| Sex | 0.58 | 0.21 – 1.59 | 0.294 |
| Charlson Comorbidity Index | 1.00 | 0.73 – 1.38 | 0.989 |
| Tumor laterality |  |  |  |
| Left sided tumor (compared to right) | 0.64 | 0.24 – 1.72 | 0.379 |
| Bilateral tumor | 0.64 | 0.05 – 9.06 | 0.740 |
| Retroperitoneal | 2.13 | 0.78 – 5.83 | 0.141 |
| Surgical Approach (compared to open) |  |  |  |
| Laparoscopic | 0.30 | 0.10 – 0.95 | 0.041 |
| Robotic | 0.12 | 0.01 – 1.27 | 0.079 |
| Hand Assisted | - | - | - |
| Planned nephrectomy | 1.11 | 0.24 –5.25 | 0.891 |
| Brier Score: 0.1740  ROC: 0.5540 |  |  |  |

**Supplementary Table 3.** Multivariable linear regression for Comprehensive Complication Index (CCI) at time of discharge for patients undergoing adrenalectomy for pheochromocytoma ≥ 7cm and ≥ 8cm

| **Multivariable Linear Regression Evaluating Factors Associated Comprehensive Complication Index (CCI) for patients with pheochromocytoma ≥ 7cm** | | | |
| --- | --- | --- | --- |
| Risk Factor | Coefficient | 95% Confidence Interval | p-value |
| Age | 0.15 | -0.05 – 0.36 | 0.157 |
| BMI | -0.04 | -0.58 – 0.50 | 0.894 |
| Sex | -3.06 | -7.80 – 1.67 | 0.204 |
| Charlson Comorbidity Index | 0.52 | -1.02 – 2.07 | 0.503 |
| Tumor laterality |  |  |  |
| Left sided tumor (compared to right) | -1.63 | -6.45 – 3.20 | 0.507 |
| Bilateral tumor | -0.23 | -11.69 – 11.23 | 0.968 |
| Retroperitoneal | 1.38 | -3.31 – 6.08 | 0.562 |
| Surgical Approach (compared to open) |  |  |  |
| Laparoscopic | -7.77 | -12.95 – -2.95 | **0.002** |
| Robotic | -8.30 | -18.36 – 1.76 | 0.105 |
| Hand Assisted | -21.79 | -55.79 – 12.22 | 0.208 |
| Planned nephrectomy | 0.98 | -8.95 – 10.91 | 0.846 |
| R^2^: 0.1070 |  |  |  |

| **Multivariable Linear Regression Evaluating Factors Associated Comprehensive Complication Index (CCI) for patients with pheochromocytoma ≥ 8cm** | | | |
| --- | --- | --- | --- |
| Risk Factor | Coefficient | 95% Confidence Interval | p-value |
| Age | 0.19 | -0.17 to 0.55 | 0.294 |
| Sex (female compared to male) | -5.31 | -13.07 to 2.45 | 0.177 |
| BMI ≥30 | -0.18 | -1.12 to 0.77 | 0.708 |
| Surgical Approach (compared to open) |  |  |  |
| Laparoscopic | -9.66 | -18.54 to -0.78 | **0.033** |
| Robotic | -13.44 | -28.15 to 1.27 | 0.073 |
| Hand Assisted | -21.44 | -60.21 to 17.33 | 0.274 |
| Charlson comorbidity index | -0.41 | -3.02 to 2.21 | 0.757 |
| Tumor laterality |  |  |  |
| Left sided tumor (compared to right) | -3.45 | -11.38 to 4.47 | 0.388 |
| Bilateral tumor (compared to right) | -7.91 | -28.18 to 12.37 | 0.757 |
| Retroperitoneal approach (compared to transperitoneal) | -0.59 | -7.62 to 6.44 | 0.868 |
| Planned nephrectomy | 1.56 | -11.02 to 14.14 | 0.806 |
| R^2^: 0.1639 |  |  |  |

**Supplementary Table 4.** Catecholamine quartiles for patients undergoing adrenalectomy for PCC

| Quartile | Total cohort  n=1,496 | PCC <6cm  n=1,017 | PCC ≥6cm  n=389 | p-value |
| --- | --- | --- | --- | --- |
| 1 | 354 (23.7) | 302 (27.3) | 52 (13.4) | <0.001 |
| 2 | 371 (24.8) | 311 (28.1) | 50 (15.4) |  |
| 3 | 376 (25.1) | 273 (24.7) | 103 (26.5) |  |
| 4 | 395 (26.4) | 221 (20.0) | 174 (44.7) |  |

Data are represented as number and percentage. Patients were ranked into quartiles based on the value of catecholamine reported of the most common outcome measure; 24 hour urine normetanephrines (n=861), plasma normetanephrines (n=538), urine adrenaline (n=64), urine metamephrines (n=27), plasma metanephrines (n=5), and urine noradrenaline (n=1).

**Supplementary Table 5.** Sensitivity and specificity for predicting complications by PCC size for optimal cutoff analysis

| **Cutoff PCC diameter (cm)** | **Sensitivity (%)** | **Specificity (%)** |
| --- | --- | --- |
| ≥ 5.4 | 47.8 | 74.9 |
| ≥ 5.6 | 44.5 | 77.7 |
| ≥ 5.8 | 43.6 | 79.0 |
| ≥ 6.0 | 42.0 | 79.8 |
| ≥ 6.2 | 38.9 | 83.3 |
| ≥ 6.4 | 37.4 | 84.6 |


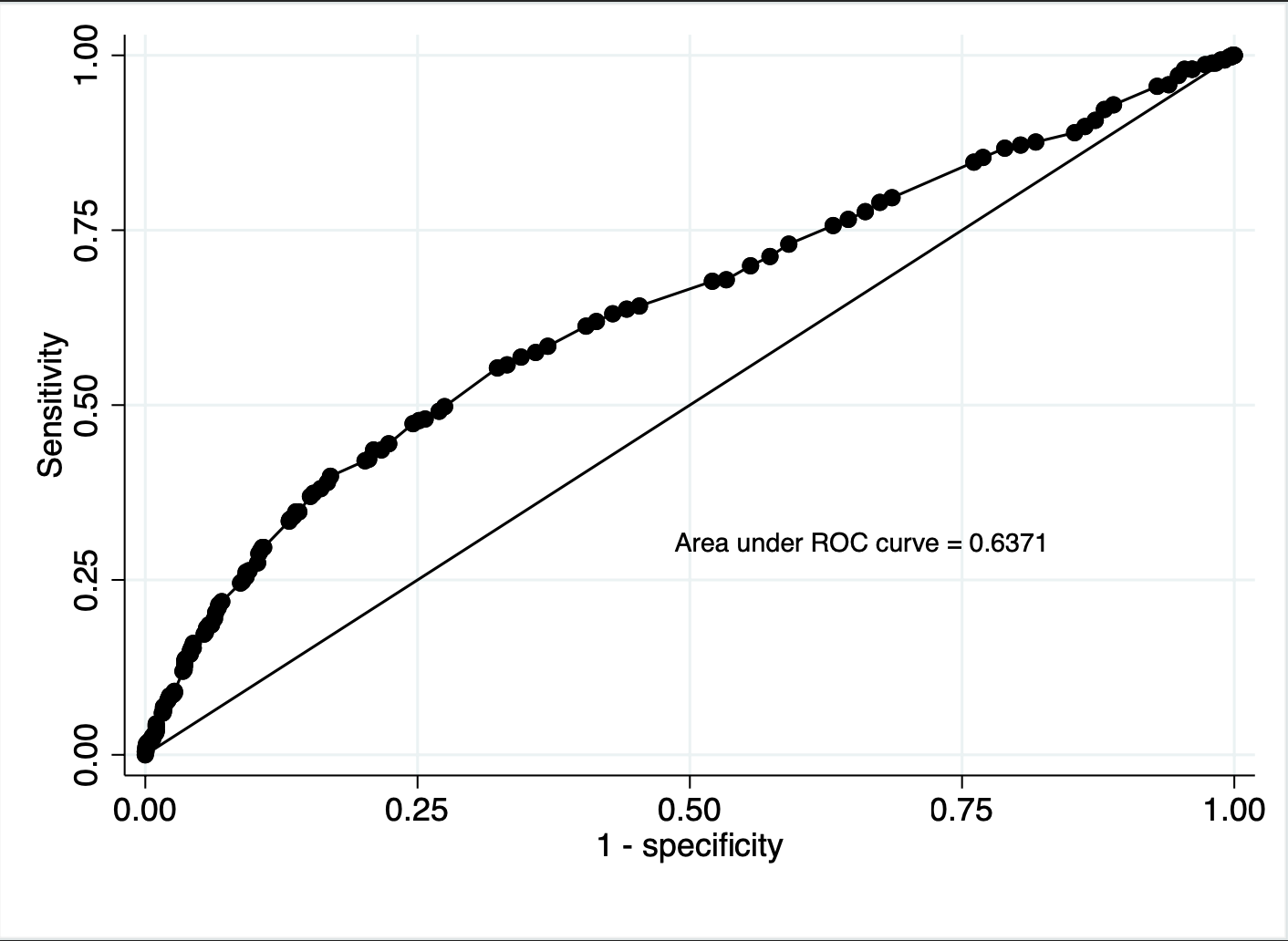


**Supplementary Figure 2.** Receiver operating characteristic (ROC) curve demonstrating linearity of the sensitivity and specificity for any complications for patients undergoing adrenalectomy for PCC.

**Supplementary Table 6.** Multivariable logistic regression for any complications at time of discharge and multivariable linear regression for Comprehensive Complication Index (CCI) for patients undergoing adrenalectomy

| **Multivariable Logistic Regression Evaluating Factors Associated with Any Complication for patients with PCC ≥ 5.8cm** | | | |
| --- | --- | --- | --- |
|  | |  | |
| Risk Factor | Odds Ratio | 95% Confidence Interval | p-value |
| Tumor Size ($\geq$5.8cm versus <5.8cm) | 2.05 | 1.53 to 2.75 | **<0.001** |
| Age | 0.99 | 0.98 to 1.00 | 0.089 |
| Sex (female compared to male) | 0.98 | 0.75 to 1.28 | 0.862 |
| BMI ≥30 | 1.00 | 0.97 to 1.03 | 0.815 |
| Surgical Approach (compared to open) |  |  |  |
| Laparoscopic | 0.31 | 0.22 to 0.45 | **<0.001** |
| Robotic | 0.39 | 0.22 to 0.70 | **0.002** |
| Hand Assisted | 0.40 | 0.04 to 4.43 | 0.457 |
| Charlson comorbidity index | 1.20 | 1.11 to 1.30 | **<0.001** |
| Tumor laterality |  |  |  |
| Left sided tumor (compared to right) | 0.99 | 0.76 to 1.31 | 0.967 |
| Bilateral tumor | 1.30 | 0.66 to 2.56 | 0.447 |
| Retroperitoneal approach (compared to transperitoneal) | 0.98 | 0.74 to 1.30 | 0.871 |
| Planned nephrectomy | 1.68 | 0.62 to 4.56 | 0.310 |
| Brier score: 0.1348  ROC: 0.6944 |  |  |  |
